# Hearing loss and postural balance performance in adults and the elderly in ELSA-Brazil

**DOI:** 10.11606/s1518-8787.2026060007332

**Published:** 2026-06-15

**Authors:** Fernanda Yasmin Odila Maestri Miguel Padilha, Vitor Martins Guesser, Camila Maia Rabelo, Renata Rodrigues Moreira, Itamar de Souza Santos, Isabela M. Bensenor, Paulo A. Lotufo, Alessandra Giannella Samelli

**Affiliations:** I Universidade de São Paulo. Faculdade de Medicina. Departamento de Fisioterapia, Fonoaudiologia e Terapia Ocupacional. São Paulo, SP, Brasil; II Universidade de São Paulo. Hospital Universitário. Serviço de Audiologia. São Paulo, SP, Brasil; III Universidade de São Paulo. Hospital Universitário. Centro de Pesquisa Clínica e Epidemiológica. São Paulo, SP, Brasil; IV Universidade de São Paulo. Faculdade de Medicina. Departamento de Clínica Médica. São Paulo, SP, Brasil

**Keywords:** Hearing Loss, Postural Balance, Ageing, Accidental Falls, Vestibular Function Tests

## Abstract

**OBJECTIVE:**

To investigate the association between hearing loss and postural balance in adults and the elderly participating in the *Estudo Longitudinal da Saúde do Adulto* (ELSA-Brazil - Longitudinal Study of Adult Health).

**METHODS:**

Cross-sectional study with data from the fourth wave (2020–2025) of ELSA-Brazil, in the center of São Paulo. Sociodemographic, clinical, and audiological variables were analyzed. Hearing loss was defined by the average of the tonal thresholds (> 25 dBHL) in different criteria: PTA (0.5 kHz-2kHz), speech-frequency PTA (0.5 kHz-4kHz), and high-frequency PTA (3 kHz-8kHz), considering the better and the worst hearing ear. Balance was assessed using the modified Romberg test, in four progressive conditions (eyes open on a firm surface, eyes closed on a firm surface, eyes open on a soft surface, and eyes closed on a soft surface), with a binary classification (pass/fail). The outcome was performance in the balance test.

**RESULTS:**

A total of 540 participants were assessed, mostly women, self-declared white, mean age of 63.1 years, mean Body Mass Index (BMI) of 28.4 kg/m^2^, with higher education and income between R$ 1,245.00 and R$ 3,319.00. The rate of balance failure was 13.3%. The prevalence of hearing loss ranged from 13.7% to 59.8%, depending on the criteria used. There was an association between hearing loss and worse performance in the balance test, especially for high-frequency PTA. In the logistic regression models, there was a significant association between the explanatory variable age and failure in the balance test. After excluding the age variable, there was an association for hearing loss, BMI, schooling, and income.

**CONCLUSION:**

There was an association between hearing loss and poorer balance performance. The highest proportion of failures was for the hearing loss criterion that included high frequencies. Age was the most relevant explanatory variable in balance test performance, in addition to hearing loss, schooling, income, and BMI, emphasizing the need for a multifactorial view of this function.

## INTRODUCTION

Hearing loss can have social, cognitive, emotional, and functional impacts^1,2^. When associated with ageing, it is called presbycusis and is the third most prevalent chronic health condition in adults and the elderly. It is estimated that around 30% of people over 50 and more than 50% of those aged 60 or over have hearing loss^3,4^.

Like hearing, balance declines progressively with age, increasing the risk of instability and falls^5^.

Studies suggest that hearing loss can interfere with vestibular function and balance, increasing the risk of falls^6^. This association can be explained by the anatomical and physiological proximity between the organs of hearing and balance, as well as the influence of common factors such as ageing, chronic diseases and ototoxic drugs^9^.

With the aim of increasing knowledge about the contribution of vestibular dysfunction to imbalance and falls in the elderly, as well as reducing the costs generated and the social impact associated with falls, the United States incorporated the modified Romberg balance test into the National Health and Nutrition Examination Survey (NHANES) for standing on firm and flexible support surfaces. This is an objective test with four conditions to assess the sensory inputs that contribute to balance: the vestibular system, vision and proprioception^10^. Based on the analysis carried out on the NHANES 2001–2004, the authors found that performance on the modified Romberg test was significantly associated with self-reported risk of falling in the previous 12 months^10^. In addition, this measure has been shown to be close to the computerized dynamic posturography test, which is one of the instruments used in the clinical diagnosis of vestibular dysfunction, since it assesses the individual ‘s ability to maintain balance when vestibular information is the only reliable sensory input. As such, the modified Romberg test has been considered promising as a screening test for balance function mediated by the vestibular system, and is a good predictor of the risk of falling during normal daily activities^10,11^.

Although there is evidence that hearing loss can increase the risk of falls in adults and the elderly, there is still no consensus on this association, since several variables can interfere with both systems, given the effect of multiple variables. Therefore, this study aims to investigate the association between hearing loss and semi-static postural balance in adults and elderly people from the *Estudo Longitudinal da Saúde do Adulto* (ELSA - Longitudinal Study of Adult Health, Brazil).

## METHODS

This is a cross-sectional study using data from the fourth wave (2020–2025) of ELSA-Brazil. ELSA-Brazil is a multicenter cohort that followed approximately 15,000 civil servants of both sexes, aged between 35 and 74 in the first wave baseline (2008–2011), linked to six public higher education and research institutions in the country. Its objectives, design, and sample profile have already been described in previous publications^12,13^.

The *Estudo Longitudinal da Saúde Auditiva do Adulto* (ELSA-A - Longitudinal Study of Adult Hearing Health) is a sub-study of ELSA-Brazil, at the São Paulo research center, with 901 participants who underwent audiological evaluation in the first wave (2008–2011). The volunteers are followed up periodically using health questionnaires and audiological tests^14^.

For the present study, all participants in the ELSA-A sub-study who were still being followed up during the fourth wave (n = 569) were considered eligible for the postural balance test. Of these, 29 did not take the balance test (criteria described below), resulting in a final sample of 540 individuals, as shown in the flowchart in Figure.

**Figure:**
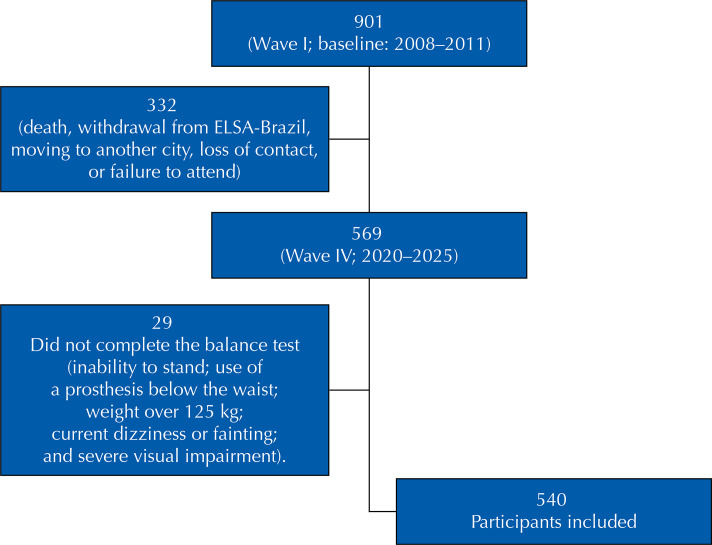
Flowchart of participants in the fourth wave of the ELSA-A.

### Procedures

The data was obtained from the ELSA-Brazil assessments and the ELSA-A sub-study, referring to the fourth wave. ELSA-Brazil collected sociodemographic information, health history, lifestyle habits, as well as laboratory and physical examinations^12,13^.

With the ELSA-A, information was obtained on hearing health, history of dizziness and falls in the last 12 months, as well as information prior to the balance test (specific exclusion criteria, namely: inability to stand, use of a prosthesis below the waist, weight over 125 kg, current presence of dizziness or fainting, and severe visual impairment). Audiological and balance assessments were also carried out.

The complete audiological assessment consisted of meatoscopy, immitanciometry (tympanometry and ipsilateral acoustic reflexes), pure tone audiometry (0.25–8 kHz by air conduction and, when necessary, 0.5–4 kHz by bone conduction) and speech tests (threshold and speech recognition index). Hearing thresholds were obtained from audiometry.

Balance screening was introduced from the fourth wave of ELSA-A, using of the modified Romberg test^15^, with four progressive conditions (condition 1: eyes open and firm surface for 15 seconds; condition 2: eyes closed and firm surface for 15 seconds; condition 3: eyes open and soft surface for 30 seconds; and condition 4: eyes closed and soft surface for 30 seconds), with the criteria for “failure” during the execution of the evaluated conditions being movement of the arms/feet, falling, the need to open the eyes or not maintaining the position for the specified time^15^. This assessment was carried out on the same day as the audiological assessment.

### Study Variables

The maintenance of the individual’s semi-static postural balance was considered the outcome (dependent) variable in this study. Performance in the Romberg test was classified as “passed” (maintaining the position for the stipulated time in all conditions) or “failed”, when they were unable to complete them.

The main independent variables in this study refer to hearing loss, defined based on the mean pure-tone hearing thresholds (Pure Tone Audiometry - PTA). Three criteria were considered: PTA (0.5, 1, and 2 kHz), speech-frequency PTA (0.5, 1, 2, and 4 kHz) and *high-frequency* PTA (3, 4, 6, and 8 kHz)^16^. For the three criteria adopted, the averages for the better and worst hearing ear were considered, totaling six criteria evaluated. The presence of hearing loss was defined based on the average of the PTA above 25 dBHL^17^.

The following were also considered as explanatory variables: gender, race, age, level of education, income, hearing difficulty, tinnitus, exposure to noise, use of hearing aids, smoking, systemic arterial hypertension (SAH), diabetes mellitus (DM), dyslipidemia (DLP), and Body Mass Index (BMI) kg/m^2^. SAH was defined by the use of antihypertensive drugs and/or systolic blood pressure ≥ 140 mmHg and/or diastolic blood pressure≥ 90 mmHg, while DM was characterized by previous diagnosis, use of medication or laboratory alterations (fasting blood glucose 126 mg/dl, glycated hemoglobin ≥ .5% or oral glucose tolerance test ≥ 200 mg/dl)^12,13^.

### Statistical Analysis

To characterize the sample, the qualitative variables were represented using absolute (n) and relative (%) frequencies. Quantitative variables were described using measures of central tendency (mean) and dispersion (standard deviation - SD).

The chi-square test of independence was used to assess associations between qualitative variables (sociodemographic, general health, and hearing) and the outcome (completion of the balance test). The Mann-Whitney test was used for continuous variables (age and BMI). The chi-square test of independence was also used to assess associations between the presence of hearing loss (better/worst ear) in the different criteria (PTA, speech-frequency PTA, and high-frequency PTA). The Mann-Whitney test was used to assess the association between age and hearing loss criteria.

For multivariate analysis, multiple logistic regression models were adjusted to investigate factors associated with the probability of passing the balance test. Initially, the following explanatory variables were included: gender, schooling, smoking, race, DLP, income, hypertension, DM, hearing difficulty, tinnitus, exposure to noise, use of hearing aids, age, BMI, and all six hearing loss criteria. The schooling, race, and income variables were recategorized as: schooling (primary and secondary/higher education), race (black, brown, white, yellow/indigenous), income (less than or equal to R$ 3,319 and greater than R$ 3,319). The variable alcohol use was not included in the model as it had many missing observations and was not associated with the final conclusion of the balance test (Table 1). The adjusted model showed multicollinearity with high variance inflation factors associated with the hearing loss criteria, which were highly correlated with each other (Table 3) and the use of hearing aids. Therefore, six logistic regression models were adjusted, one for each hearing loss criterion, including the other explanatory variables, except for the use of hearing aids. Variables were selected using the backward method. Odds ratio and respective 95% confidence intervals were estimated. The analyses were carried out using Minitab 21 and R (version 4.3.1), with a significance level of 5% (p < 0.05).

**Table 1:** Descriptive statistics for sociodemographic variables, clinical conditions, and lifestyle habits, by final balance test conclusion.

Variable	Variable Total	Balance test		Statistical value (g.l.)	p-value
		Failed	Passed		
Sex, n (%)					
Female	294 (54.4)	34 (11.6)	260 (88.4)	1.75 (1)	0.186^a^
Male	246 (45.6)	38 (15.5)	208 (84.5)		
Schooling, n (%)					
Elementary	46 (8.5)	11 (23.9)	35 (76.1)		
High school	231 (42.8)	25 (10.8)	206 (89.2)	5.75 (2)	0.057^a^
Higher education	263 (48.7)	36 (13.7)	227 (86.3)		
Alcohol use, n (%)					
Never used	75 (16.1)	8 (10.7)	67 (89.3)		
Former user	63 (13.6)	8 (12.7)	55 (87.3)	0.43 (2)	0.809^a^
User	327 (70.3)	44 (13.5)	283 (86.5)		
Smoker, n (%)					
Never smoked	324 (61.4)	35 (10.8)	289 (89.2)		
Former smoker	157 (29.7)	24 (15.3)	133 (84.7)	2.21 (2)	0.330^a^
Smoker	47 (8.9)	7 (14.9)	40 (85.1)		
Race, n (%)					
Black	73 (13.7)	4 (5.5)	69 (94.5)		
Brown	109 (20.3)	13 (11.9)	96 (88.1)		
White	316 (58.8)	45 (14.2)	271 (85.8)	5.42 (4)	0.170^a^
Yellow	33 (6.1)	6 (18.2)	27 (81.8)		
Indigenous	6 (1.1)	2 (33.3)	4 (66.7)		
Dyslipidemia, n (%)					
No	234 (46.2)	25 (10.7)	209 (89.3)	2.09 (1)	0.148^a^
Yes	273 (53.8)	41 (15.0)	232 (85.0)		
Income (R$), n (%)					
Less than 1,245	169 (31.4)	23 (13.6)	146 (86.4)		
Between 1,245 and 3,319	244 (45.4)	23 (9.4)	221 (90.6)	9.24 (2)	0.010^a^
Greater than 3,319	125 (23.2)	26 (20.8)	99 (79.2)		
Hypertension, n (%)					
No	263 (52.0)	29 (11.0)	234 (89.0)	1.62 (1)	0.203^a^
Yes	243 (48.0)	36 (14.8)	207 (85.2)		
Diabetes, n (%)					
No	375 (71.6)	43 (11.5)	332 (88.5)	1.53 (1)	0.217^a^
Yes	149 (28.4)	23 (15.4)	126 (84.6)		
Hearing difficulty, n (%)					
No	344 (63.8)	39 (11.3)	305 (88.7)	3.36 (1)	0.067^a^
Yes	195 (36.2)	33 (16.9)	162 (83.1)		
Tinnitus, n (%)					
No	327 (60.6)	44 (13.5)	283 (86.5)	0.01 (1)	0.917^a^
Yes	213 (39.4)	28 (13.2)	185 (86.8		
Exposure to noise, n (%)					
No	326 (60.4)	48 (14.7)	278 (85.3)	1.38 (1)	0.241^a^
Yes	214 (39.6)	24 (11.2)	190 (88.8)		
Use of hearing aids, n (%)					
No	520 (96.7)	65 (12.5)	455 (87.5)	10.45 (1)	0.001^a^
Yes	18 (3.3)	7 (38.9)	11 (61.1)		
Age (years) - mean (SD)	63.1 (8.7)	71.3 (8.7)	61.9 (8.0)	60.91 (1)	< 0.001^b^
BMI (kg/m^2^) - mean (SD)	28.4 (5.2)	27.0 (4.4)	28.5 (5.2)	5.73 (1)	0.017^b^

SD: standard deviation; g.l.: degrees of freedom.

Note: missing data: alcohol use (75 observations), smoking (12 observations), race (3 observations), dyslipidemia (33 observations), income (2 observations), hypertension (34 observations), diabetes (16 observations), BMI (19 observations), hearing difficulty (1 observation), and use of hearing aids (2 observations).

Statistical significance. A 5% significance level was adopted.

^a^ Pearson’s chi-squared test.

^b^Mann-Whitney test.

**Table 3:** Joint frequency distribution of hearing loss in the better/worst eara.

Ear - criterion	Ear - criterion							n (%) of individuals with hearing loss according to each criterion
		No		Yes		p-value	Statistical value (g.l.)	
		n	%	n	%	(c^2^)		
	Better ear - PTA							Better ear - PTA
Better ear-speech-frequency PTA	No	432	92.7	2	2.7		327.88 (1)	74 (13.7)
	Yes	34	7.3	72	97.3	< 0.001		
	Better ear - PTA							Better ear - high-frequency PTA
Better ear - high-frequency PTA	No	290	62.2	2	2.7		91.12 (1)	248 (45.9)
	Yes	176	37.8	72	97.3	< 0.001		
	Worst ear - PTA							Worst ear - PTA
Worst ear - speech-frequency PTA	No	375	89.3	4	3.3		329.52 (1)	120 (22.2)
	Yes	45	10.7	116	96.7	< 0.001		
	Worst ear - PTA							Worst ear - high-frequency PTA
Worst ear - high-frequency PTA	No	215	51.2	2	1.7		95.23 (1)	323 (59.8)
	Yes	205	48.8	118	98.3	< 0.001		
	Better ear - speech-frequency PTA							Better ear - speech-frequency PTA
Better ear - high-frequency PTA	No	292	67.3	0	0.0		155.29 (1)	106 (19.6)
	Yes	142	32.7	106	100.0	< 0.001		
	Worst ear - speech-frequency PTA							Worst ear - speech-frequency PTA
Worst ear - high-frequency PTA	No	217	57.3	0	0.0		154.11 (1)	161 (29.8)
	Yes	162	42.7	161	100.0	< 0.001		

PTA: pure tone audiometry; g.l.: degrees of freedom.

^a^ Distribution of hearing loss in the better/worst ear: PTA and hearing loss in the better/worst ear - speech-frequency - PTA and high-frequency - PTA and joint frequency distribution of hearing loss in the better/worst ear -speech-frequency - PTA and hearing loss in the better/worst ear -high-frequency - PTA; number (percentage) of individuals, among the 540 studied, who had hearing loss in the better and worst ear under PTA, speech-frequency PTA, and high-frequency PTA. Statistical significance: 5% significance level and Pearson’s chi-squared test.

### Ethical Aspects

This study was approved by the Ethics Committee for the Analysis of Research Projects of Hospital das Clínicas da Faculdade de Medicina da Universidade de São Paulo (CAAE 15171019.7.0000.0065).

## RESULTS

Among the 540 individuals, there was a higher prevalence of females (54.4%), an average age of 63.1 years (8.7) and an average BMI of 28.4 kg/m^2^ (5.2). Regarding schooling, 48.6% had higher education. As for clinical and audiological conditions, 53.8% had SLD, 48% had SAH and 28.4% had DM, 70.3% were alcohol users, 36.2% reported hearing difficulty, 39.4% had tinnitus, 39.6% reported exposure to noise, and only 3.3% used hearing aids. The failure rate in the balance test was 13.3% (n = 72). The bivariate analysis showed a statistically significant association between completion of the balance test and the variables age (p < 0.001), BMI (p = 0.017), income (p = 0.010), and use of hearing aids (p = 0.001) (Table 1).

Table 2 shows the association between hearing loss and the final conclusion of the balance test. There was a significant association between the presence of hearing loss in both the better and worst ear for the PTA, speech-frequency PTA, and high-frequency PTA criteria and the balance test (p < 0.001 in all cases), with a higher proportion of balance test failures for individuals with hearing loss.

**Table 2:** Joint frequency distribution of hearing loss and final balance test conclusion.

Hearing loss	Balance test				Statistical value (g.l.)	p-value (c)
	Failed		Passed			
	n	%	n	%		
Better ear - PTA						
No	52	11.2	414	88.8	13.92 (1)	< 0.001^a^
Yes	20	27.0	54	73.0		
Worst ear - PTA						
No	43	10.2	377	89.8	15.67 (1)	< 0.001^a^
Yes	29	24.2	91	75.8		
Better ear - speech-frequency PTA						
No	42	9.7	392	90.3	25.57 (1)	< 0.001^a^
Yes	30	28.3	76	71.7		
Worst ear - speech-frequency PTA						
No	36	9.5	343	90.5	16.18 (1)	< 0.001^a^
Yes	36	22.4	125	77.6		
Better ear - high-frequency PTA						
No	22	7.5	270	92.5	18.50 (1)	< 0.001^a^
Yes	50	20.2	198	79.8		
Worst ear - high-frequency PTA						
No	15	6.9	202	93.1	12.94 (1)	< 0.001^a^
Yes	57	17.7	266	82.3		

PTA: pure tone audiometry; g.l.: degrees of freedom.

^a^ Statistical significance. 5% significance level and Pearson’s chi-squared test.

Regarding the criteria for hearing loss, it was found that, considering the better ear, hearing loss was identified in 13.7% of individuals by PTA, 19.6% by speech-frequency PTA and 45.9% by high-frequency PTA. In the worst ear, the prevalence of hearing loss was 22.2% by PTA, 29.8% by speech-frequency PTA, and 59.8% by high-frequency PTA (Table 3).

After applying the backward variable selection method, the final six adjusted logistic regression models only included the age variable. It was found that the chance of an individual passing the balance test decreases by 11.1% ([1–0.889] x 100%) with each one-year increase in age (Table 5). It is worth noting that although age is associated with hearing loss (Table 4), no adjusted logistic regression model showed multicollinearity between age and hearing loss.

**Table 5:** Results of the final adjusted logistic regression model.

Term	Estimate	Standard Error	p-value	Odds ratio	95%CI
Constant	9.720	1.070	< 0.001^a^		
Age	-0.118	0.015	< 0.001^a^	0.889	(0.862–0.916)

95%CI: 95% confidence interval.

^a^ Statistical significance. 5% significance level and multiple logistic regression model adjusted by the backward method.

**Table 4:** Mean (standard deviation) of age for hearing loss in the better/worst ear under PTA, speech-frequency PTA, and high-frequency PTA.

Hearing loss	No	Yes	Statistical value (g.l.)	p-value
Better ear - PTA	61.7 (7.8)	72.1 (9.2)	68.33 (1)	< 0.001^a^
Worst ear - PTA	61.3 (7.5)	69.5 (9.6)	65.85 (1)	< 0.001^a^
Better ear - speech-frequency PTA	61.1 (7.3)	71.6 (8.9)	97.99 (1)	< 0.001^a^
Worst ear - speech-frequency PTA	60.6 (7.1)	69.2 (9.1)	98.52 (1)	< 0.001^a^
Better ear - high-frequency PTA	59.3 (6.5)	67.7 (8.8)	119.92 (1)	< 0.001^a^
Worst ear - high-frequency PTA	58.5 (6.2)	66.2 (8.8)	101.65 (1)	< 0.001^a^

PTA: pure tone audiometry; g.l.: degrees of freedom.

^a^ Statistical significance. 5% significance level and Mann-Whitney test.

To deepen the analysis of the association between the outcome and the presence of hearing loss for the six criteria, age was categorized into four age groups (up to 59 years, 60 to 69 years, 70 to 79 years and 80 to 89 years). Then, for each age group, six logistic regression models were fitted, one for each hearing loss criterion, including the other explanatory variables. For all the models, after applying the backward variable selection method, no explanatory variable showed significance.

## DISCUSSION

Our findings showed an association between hearing loss and worse performance in the semi-static postural balance test in adults and the elderly in ELSA-Brazil. In addition, we found that the proportion of failures in the balance test was significantly higher for the hearing loss criterion that included high frequencies when calculating the mean hearing thresholds. In addition, the explanatory variables age, income, and BMI showed an association with worse performance in the balance test in the bivariate analyses. However, after adjustment, the only variable that remained associated with the outcome was age.

In the analysis considering the six hearing loss criteria and performance in the balance test, a higher prevalence of failure was observed for all of them in individuals with hearing loss. These findings are in line with evidence that hearing loss can affect spatial orientation, the detection of environmental sound cues and, consequently, increase the risk of falls^18^. This is because hearing impairment can reduce the capacity for sensory integration between the auditory and vestibular systems, impairing postural stability^6^.

In the study by Lin et al.^18^, using data from NHANES^15^, the relationship between hearing loss, defined by the average audiometric thresholds at frequencies of 0.5, 1, 2, and 4 kHz, and self-reported falls in adults aged 40 to 69 was analyzed. The results showed that for every 10 dB increase in hearing loss, there was a 1.4-fold increase in the likelihood of reporting a fall in the previous 12 months, even after adjusting for multiple confounding factors.

In a 10-year longitudinal follow-up, Jansen et al.^19^ investigated the association between speech-in-noise and recurrent falls. The study included adults aged 40 and over and showed that poorer performance on the speech-in-noise test was associated with an approximately two-fold increased risk of recurrent falls, especially in participants with obesity and higher hearing thresholds. The authors suggested that difficulty processing speech signals in acoustically complex contexts can compromise spatial orientation and increase vulnerability to falls, reinforcing the importance of considering auditory function at frequencies relevant to speech perception in noisy environments. Moreover, hearing loss often coexists with vestibular dysfunction and chronic comorbidities, such as DM cardiovascular disease, which can potentiate postural instability^20^.

A comparative analysis of the hearing loss criteria showed different prevalences of hearing loss (ranging from 13.7% to 59.8%). Different hearing loss criteria are used in the literature, with different combinations of frequencies and/or ears, as well as cut-off criteria at different hearing thresholds, which can lead to different hearing loss classifications, prevalences and magnitudes of the associations found^16,21^.

The choice of hearing loss criterion should be directly related to the objectives and outcomes being investigated. For example, the studies by Yévenes-Briones et al.^21^and Lin et al.^16^ used three hearing loss criteria, in a similar way to the present study, allowing different dimensions of hearing to be assessed. The PTA is used to represent hearing sensitivity in the middle frequencies, while the speech-frequency PTA adds the frequency of 4,000 Hz, which is important for speech intelligibility. High-frequency PTA focuses on higher frequencies, which are often the first to be affected in age-related or noise-induced hearing loss^22^.

Putter-Katz et al.^23^ and Schubert et al.^24^ identified an association between hearing loss and postural instability. Putter-Katz et al.^23^ showed that hearing loss at high frequencies contributes to greater instability and risk of falls, suggesting that degradation of the auditory pathway affects compensatory balance mechanisms. Schubert et al.^24^ pointed out that hearing loss, regardless of frequency range, is related to worse scores in vestibular and gait tests, reinforcing the hypothesis that hearing acts as an additional sensory component in postural control. These findings reinforce the importance of audiological assessment, including high frequencies in the criteria for classifying hearing loss, when the aim is to assess balance and the risk of falls in the elderly.

For this reason, in the case of the present study, which assessed older adults, we used criteria that reflected age-related hearing loss, which begins with the higher frequencies^25,26^. In fact, we found higher prevalence of hearing loss for the high-frequency PTA criterion, and also for this criterion, there was a higher proportion of failures in the balance test.

As for the association between performance in the balance test and the explanatory variables, including hearing loss, as well as the sociodemographic and clinical variables, after applying the backward variable selection method , the final adjusted logistic regression models only included the age variable, indicating that age was the only one that showed significance, as well as the chance of an individual passing the balance test decreasing by 11.1% with each one-year increase in age. It is known that ageing leads to a progressive decline in physical and functional capacities, with direct repercussions on balance and mobility, which can reduce functional performance in balance and increase the risk of falls in this population. Vermisso et al.^27^ assessed functional status and balance capacity in healthy elderly people and found an association between balance and functional status in the elderly, so that deficits in one are reflected in the other. Other authors^28,29^have emphasized that the decline in functional status due to ageing can be attributed to changes in balance.

It should be mentioned that, initially, in the bivariate analysis, in addition to age and hearing loss, the variables income and BMI showed an association with the outcome. However, in the adjusted analyses, the only variable that maintained an association with balance was age.

Previous studies^30,31^ found that low income and less schooling were associated with falls in the elderly, suggesting the influence of sociodemographic conditions on the vulnerability of this population. These results indicate that different groups can be disproportionately affected when it comes to falls/postural stability; therefore, these variables should be considered in this type of study, as well as when comparing different studies. It is important to emphasize that the sample evaluated here is different, since all of them are active or retired civil servants and therefore have better financial conditions and more access to health services than the Brazilian population in general^13^. In addition, almost half of the participants had higher education, a proportion well above the national average^32^. As suggested in a previous study^33^, higher levels of education can lead to greater knowledge about health, healthier lifestyle habits and more timely access to health services.

Regarding BMI, the association initially found in the bivariate analysis was between lower BMI and failure in the balance test. A previous study^34^ found that a higher BMI was associated with a lower chance of falls in a group of elderly women and the authors suggested that a greater amount of body tissue could have a protective effect, either through greater cushioning in the event of an impact or through the positive relationship with lean mass, which is important for preserving functional capacity. In contrast, another study^35^ observed that obese individuals had a higher risk of falls compared to those with a healthy weight, highlighting that the former had a higher prevalence of chronic diseases, use of multiple medications and poorer quality of life. Third study^36^pointed out that low BMI, which is a marker of malnutrition in the elderly, was strongly associated with a higher risk of falls and fractures, mainly due to the loss of lean mass and sarcopenia. Thus, the authors emphasized that, in the elderly, higher BMI cut-off points (24 kg/m^2^–27kg/m^2^), when compared to younger adults, are considered appropriate and can play a protective role^36^.

Aiming to deepen the analysis between performance in the balance test and hearing loss, considering that the only variable that remained associated with the outcome was age, we categorized this variable into four age groups and, for each of them, we adjusted the six logistic regression models, one for each hearing loss criterion, which included the other explanatory variables using the backward method. However, there was no statistical significance for any of the explanatory variables, including hearing loss, in any of the models, confirming that the outcome is explained only by the age variable. This finding emphasizes the weight of the age variable in balance performance, overriding the other variables, similar to that found for hearing loss^16^. One possible explanation for these results is that some of these variables (sociodemographic and clinical) may have a weaker association (as seen in the first analyses of this study), but their effects are masked by stronger risk factors, such as age^16^.

In a systematic review and meta-analysis on the subject, the authors emphasize that it is unclear whether there is a real causal association between hearing loss and changes in balance or just the coexistence of age-related changes, which leads to impairment of both systems. The study found that age-related hearing loss was associated with impaired balance and mobility; however, the authors pointed out that part of the deficit observed reflected residual confounding factors related to age. Thus, they suggested that although chronological age remains a determining factor in balance-related outcomes, age-related hearing loss contributes an additional and modifiable risk through sensory and cognitive-motor pathways, and that rehabilitation can offer clinically significant gains in postural control within multimodal fall prevention strategies^37^.

As limitations of the study, some aspects should be considered. Firstly, the fact that the sample studied was part of ELSA-Brazil should be considered, as this is a group with greater access to health services, greater awareness of self-care and possibly better adherence to preventive practices, which may have influenced the outcomes observed. Furthermore, due to the cross-sectional design, it is not possible to establish causal relationships or temporality between hearing loss and balance performance. It should also be mentioned that the exclusive use of the modified Romberg test to assess balance does not replace more detailed instrumental assessments and therefore limits the assessment of finer components of postural control, which may reduce the identification of more subtle associations.

Among the strengths of this study is the use of a large sample from ELSA-Brazil/ELSA-A, which uses standardized protocols and objective clinical and laboratory tests (including audiological assessment and balance screening), which strengthens the validity of the results obtained. In addition, this study is innovative in the Brazilian context.

In summary, age proved to be the main variable associated with postural balance performance in adults and the elderly in ELSA-A. Hearing loss was associated with balance, particularly when high frequencies were considered. Sociodemographic and clinical variables, such as income and BMI, seem to influence balance performance in a more complex and indirect way. These findings reinforce the need for integrated and multifactorial approaches to fall prevention, incorporating hearing assessment and balance screening into the healthcare of adults and the elderly.

## Data Availability

The data are available on request from the corresponding author.
