# Supplementary material for: Hearing loss and postural balance performance in adults and the elderly in ELSA-Brazil
Source: Rev Saude Publica. 2026 Jun 15;60:e22. doi: 10.11606/s1518-8787.2026060007332 (PMC13268289; doi:10.11606/s1518-8787.2026060007332)
Supplement: Translation

# Translation: Perda auditiva e desempenho no equilíbrio postural em adultos e idosos do ELSA-Brasil

**Authors:** Fernanda Yasmin Odila Maestri Miguel Padilha, Vitor Martins Guesser, Camila Maia Rabelo, Renata Rodrigues Moreira, Itamar de Souza Santos, Isabela M. Bensenor, Paulo A. Lotufo, Alessandra Giannella Samelli

**Affiliations:** I Universidade de São Paulo. Faculdade de Medicina. Departamento de Fisioterapia, Fonoaudiologia e Terapia Ocupacional. São Paulo, SP, Brasil; II Universidade de São Paulo. Hospital Universitário. Serviço de Audiologia. São Paulo, SP, Brasil; III Universidade de São Paulo. Hospital Universitário. Centro de Pesquisa Clínica e Epidemiológica. São Paulo, SP, Brasil; IV Universidade de São Paulo. Faculdade de Medicina. Departamento de Clínica Médica. São Paulo, SP, Brasil

**Keywords:** Perda Auditiva, Equilibro Postural, Envelhecimento, Acidentes por Quedas, Testes de Função Vestibular

## Abstract

**OBJETIVO:**

Investigar a associação entre perda auditiva e equilíbrio postural em adultos e idosos participantes do Estudo Longitudinal da Saúde do Adulto (ELSA-Brasil).

**MÉTODOS:**

Estudo transversal com dados da quarta onda (2020–2025) do ELSA-Brasil, no centro de São Paulo. Foram analisadas variáveis sociodemográficas, clínicas e audiológicas. A perda auditiva foi definida pela média dos limiares tonais (> 25 dBNA) em diferentes critérios: PTA (0,5 kHz–2 kHz), *speech-frequency* PTA (0,5 kHz–4 kHz) e *high-frequency* PTA (3 kHz–8 kHz), considerando melhor e pior orelha. O equilíbrio foi avaliado pelo teste de Romberg modificado, em quatro condições progressivas (olhos abertos em superfície firme, olhos fechados em superfície firme, olhos abertos em superfície maleável e olhos fechados em superfície maleável), com classificação binária (passou/falhou). O desfecho foi o desempenho no teste de equilíbrio.

**RESULTADOS:**

Foram avaliados 540 participantes, em sua maioria mulheres, autodeclarados brancos, média de idade de 63,1 anos, Índice de Massa Corporal (IMC) médio de 28,4 kg/m^2^, com escolaridade superior e renda entre R$ 1.245,00 e R$ 3.319,00. A taxa de falha no equilíbrio foi de 13,3%. A prevalência de perda auditiva variou de 13,7% a 59,8%, dependendo do critério adotado. Observou-se associação entre perda auditiva e pior desempenho no teste de equilíbrio, sobretudo para *high-frequency* PTA. Nos modelos de regressão logística, houve associação significativa entre a variável explicativa idade e falha no teste de equilíbrio. Após exclusão da variável idade, houve associação para perda auditiva, IMC, escolaridade e renda.

**CONCLUSÃO:**

Houve associação entre perda auditiva e pior desempenho no equilíbrio. A maior proporção de falhas foi para o critério de perda auditiva que incluiu as frequências altas. A idade foi a variável explicativa mais relevante no desempenho no teste de equilíbrio, além da perda auditiva, escolaridade, renda e IMC, enfatizando a necessidade da visão multifatorial dessa função.

## INTRODUÇÃO

A perda auditiva pode causar impactos sociais, cognitivos, emocionais e funcionais^1,2^. Quando associada ao envelhecimento, denomina-se presbiacusia, sendo a terceira condição crônica de saúde mais prevalente em adultos e idosos. Estima-se que cerca de 30% das pessoas com mais de 50 anos e mais de 50% dos que têm 60 anos ou mais possuam perda auditiva^3,4^.

Assim como a audição, o equilíbrio sofre declínio progressivo com o envelhecimento, aumentando o risco de instabilidade e quedas^5^.

Estudos sugerem que a perda auditiva pode interferir na função vestibular e no equilíbrio, aumentando o risco de quedas^6^. Essa associação pode ser explicada pela proximidade anatômica e fisiológica entre os órgãos da audição e do equilíbrio, além da influência de fatores comuns, como o próprio envelhecimento, doenças crônicas e fármacos ototóxicos^9^.

Visando aprofundar os conhecimentos sobre a contribuição da disfunção vestibular para o desequilíbrio e as quedas em idosos, assim como a diminuição dos custos gerados e do impacto social associados às quedas, os Estados Unidos incorporaram à *National Health and Nutrition Examination Survey* (NHANES) o teste de Romberg modificado para equilíbrio em pé sobre superfícies de apoio firmes e flexíveis. Este é um teste objetivo com quatro condições para avaliar as entradas sensoriais que contribuem para o equilíbrio: o sistema vestibular, a visão e a propriocepção^10^. Com base na análise realizada referente ao NHANES 2001–2004, os autores verificaram que o desempenho no teste de Romberg modificado foi significativamente associado ao risco de queda autorrelatado nos 12 meses anteriores^10^. Além disso, esta medida demonstrou aproximar-se do teste de posturografia dinâmica computadorizada, que é um dos instrumentos usados no diagnóstico clínico da disfunção vestibular, já que avalia a capacidade do indivíduo em manter o equilíbrio quando a informação vestibular é a única entrada sensorial confiável. Sendo assim, o teste de Romberg modificado foi considerado promissor como um teste de triagem da função de equilíbrio mediada pelo sistema vestibular, sendo um bom preditor do risco de queda durante as atividades diárias normais^10,11^.

Apesar de existirem evidências de que a perda auditiva pode aumentar o risco de quedas em adultos e idosos, ainda não há consenso sobre essa associação, uma vez que diversas variáveis podem interferir em ambos os sistemas, dado o efeito de múltiplas variáveis. Desta forma, este estudo tem como objetivo investigar a associação entre perda auditiva e equilíbrio postural semiestático em adultos e idosos do Estudo Longitudinal da Saúde do Adulto (ELSA - Brasil).

## MÉTODOS

Estudo transversal, que utilizou dados da quarta onda (2020–2025) do ELSA-Brasil. O ELSA-Brasil constitui uma coorte multicêntrica que acompanha aproximadamente 15 mil servidores públicos, de ambos os sexos, com idades entre 35 e 74 anos na primeira onda (Linha de base: 2008–2011), vinculados a seis instituições públicas de ensino superior e pesquisa no país. Seus objetivos, desenho e perfil da amostra já foram descritos em publicações anteriores^12,13^.

O Estudo Longitudinal da Saúde Auditiva do Adulto (ELSA-A) é um subestudo do ELSA-Brasil, no centro de pesquisa de São Paulo, com 901 participantes que realizaram avaliação audiológica na primeira onda (2008–2011). Os voluntários são acompanhados periodicamente por meio de questionários de saúde e exames audiológicos^14^.

Para o presente estudo, todos os participantes do subestudo ELSA-A, que ainda estavam em seguimento durante a quarta onda (n = 569), foram considerados elegíveis para o teste de equilíbrio postural. Destes, 29 não realizaram o teste de equilíbrio (critérios descritos a seguir), resultando em uma amostra final de 540 indivíduos, como mostra o fluxograma apresentado na Figura.

**Figura:** Fluxograma participantes da quarta onda do ELSA-A.

### Procedimentos

Os dados foram obtidos a partir das avaliações do ELSA-Brasil e do subestudo ELSA-A, referentes à quarta onda. A partir do ELSA-Brasil, foram coletadas informações sociodemográficas, histórico de saúde, hábitos de vida, além de exames laboratoriais e físicos^12,13^.

Já pelo ELSA-A, foram obtidas informações sobre saúde auditiva, histórico sobre tontura e quedas nos últimos 12 meses, bem como informações prévias à aplicação do teste de equilíbrio (critérios de exclusão específicos, a saber: incapacidade para ficar em pé, uso de prótese abaixo da cintura, peso superior a 125 kg, presença atual de tontura ou desmaio e deficiência visual severa). Foram ainda realizadas as avaliações audiológica e de equilíbrio.

A avaliação audiológica completa é composta por meatoscopia, imitanciometria (timpanometria e reflexos acústicos ipsilaterais), audiometria tonal liminar (0,25–8 kHz por via aérea e, quando necessário, 0,5–4 kHz por via óssea) e logoaudiometria (limiar e Índice de Reconhecimento de Fala). A partir da audiometria, foram obtidos os limiares auditivos.

A triagem do equilíbrio foi introduzida a partir da quarta onda do ELSA-A, por meio do teste de Romberg modificado^15^, com quatro condições progressivas (condição 1: olhos abertos e superfície firme, durante 15 segundos; condição 2: olhos fechados e superfície firme durante 15 segundos; condição 3: olhos abertos e superfície maleável, durante 30 segundos; e condição 4: olhos fechados e superfície maleável, durante 30 segundos), tendo como critérios de “falha” durante a execução das condições avaliadas, a movimentação dos braços/pés, queda, necessidade de abrir os olhos ou a não manutenção da posição pelo tempo determinado^15^. Esta avaliação foi realizada no mesmo dia da avaliação audiológica.

### Variáveis do Estudo

A manutenção do equilíbrio postural semiestático do indivíduo foi considerada como variável de desfecho (dependente) neste estudo. O desempenho no teste de Romberg foi classificado como “passou” (manutenção da posição pelo tempo estipulado em todas as condições) ou “falhou”, quando não conseguiu completá-las.

As principais variáveis independentes deste estudo referem-se à perda auditiva, definida a partir das médias dos limiares auditivos de tons puros (PTA). Foram considerados três critérios: PTA (0,5; 1 e 2 kHz), *speech-frequency* PTA (0,5; 1; 2 e 4 kHz) e *high-frequency* PTA (3; 4; 6; e 8 kHz)^16^. Para os três critérios adotados, foram consideradas as médias para melhor e para a pior orelha, totalizando seis critérios avaliados. A presença de perda auditiva foi definida a partir da média dos limiares auditivos tonais acima de 25 dBNA^17^.

Também foram consideradas como variáveis explicativas: sexo, raça, idade, nível de escolaridade, renda, dificuldade para ouvir, zumbido, exposição a ruído, uso de prótese auditiva, tabagismo, hipertensão arterial sistêmica (HAS), diabetes mellitus (DM), dislipidemia (DLP) e Índice de Massa Corporal (IMC) kg/m^2^. A HAS foi definida pelo uso de anti-hipertensivos e/ou valores de pressão arterial sistólica ≥ 140 mmHg e/ou diastólica ≥ 90 mmHg, enquanto a DM foi caracterizada por diagnóstico prévio, uso de medicamentos ou alterações laboratoriais (glicemia de jejum ≥ 126 mg/dl, hemoglobina glicada ≥ 6,5% ou teste oral de tolerância à glicose ≥ 200 mg/dl)^12,13^.

### Análise Estatística

Para caracterizar a amostra, as variáveis qualitativas foram representadas por meio de frequências absolutas (n) e relativas (%). As variáveis quantitativas foram descritas por meio de medidas de tendência central (média) e de dispersão (desvio padrão - DP).

Para avaliar associações entre variáveis qualitativas (sociodemográficas, saúde geral e auditiva) e o desfecho (conclusão do teste de equilíbrio), utilizou-se o teste qui-quadrado de independência. Para variáveis contínuas (idade e IMC), aplicou-se o teste de Mann-Whitney. Para avaliar associações entre a presença de perda auditiva (melhor/pior orelha) nos diferentes critérios (PTA, *speech-frequency* PTA e *high-frequency* PTA), também foi aplicado o teste qui-quadrado de independência. Para avaliar associação entre a idade e os critérios de perda auditiva foi aplicado o teste de Mann-Whitney.

Para análise multivariada, foram ajustados modelos de regressão logística múltipla, com o objetivo de investigar fatores associados à probabilidade de aprovação (“passou”) no teste de equilíbrio. Inicialmente, foram incluídas as variáveis explicativas: sexo, escolaridade, fumante, raça, DLP, renda, hipertensão, DM, dificuldade para ouvir, zumbido, exposição a ruído, uso de prótese auditiva, idade, IMC e todos os seis critérios de perdas auditivas. As variáveis escolaridade, raça e renda foram recategorizadas como: escolaridade (fundamental e médio/superior), raça (preta, parda, branca, amarela/indígena), renda (menor ou igual a 3.319 reais e maior que 3.319 reais). A variável uso de álcool não foi incluída no modelo, pois apresentou muitas observações faltantes e não se mostrou associada à conclusão final do teste de equilíbrio (Tabela 1). O modelo ajustado exibiu multicolinearidade com fatores de inflação de variância altos associados aos critérios de perda auditiva, que se mostraram muito correlacionados entre si (Tabela 3) e ao uso de prótese auditiva. Dessa forma, foram ajustados seis modelos de regressão logística, um para cada critério de perda auditiva, incluídas as demais variáveis explicativas, com exceção do uso de prótese. A seleção de variáveis foi realizada pelo método *backward*. Foram estimadas razões de chances (*odds ratios*) e respectivos intervalos de 95% de confiança. As análises foram realizadas nos programas Minitab 21 e R (versão 4.3.1), adotando-se nível de significância de 5% (p < 0,05).

**Tabela 1:** Estatísticas descritivas das variáveis sociodemográficas, condições clínicas e hábitos de vida, por conclusão final do teste de equilíbrio.

Variável	Total	Teste de equilíbrio		Valor da estatística (g.l.)	Valor-p
		Falhou	Passou		
Sexo, n (%)					
Feminino	294 (54,4)	34 (11,6)	260 (88,4)	1,75 (1)	0,186^a^
Masculino	246 (45,6)	38 (15,5)	208 (84,5)		
Escolaridade, n (%)					
Fundamental	46 (8,5)	11 (23,9)	35 (76,1)		
Médio	231 (42,8)	25 (10,8)	206 (89,2)	5,75 (2)	0,057^a^
Superior	263 (48,7)	36 (13,7)	227 (86,3)		
Uso de álcool, n (%)					
Nunca usou	75 (16,1)	8 (10,7)	67 (89,3)		
Ex-usuário	63 (13,6)	8 (12,7)	55 (87,3)	0,43 (2)	0,809^a^
Usuário	327 (70,3)	44 (13,5)	283 (86,5)		
Fumante, n (%)					
Nunca fumou	324 (61,4)	35 (10,8)	289 (89,2)		
Ex-fumante	157 (29,7)	24 (15,3)	133 (84,7)	2,21 (2)	0,330^a^
Fumante	47 (8,9)	7 (14,9)	40 (85,1)		
Raça, n (%)					
Preta	73 (13,7)	4 (5,5)	69 (94,5)		
Parda	109 (20,3)	13 (11,9)	96 (88,1)		
Branca	316 (58,8)	45 (14,2)	271 (85,8)	5,42 (4)	0,170^a^
Amarela	33 (6,1)	6 (18,2)	27 (81,8)		
Indígena	6 (1,1)	2 (33,3)	4 (66,7)		
Dislipidemia, n (%)					
Não	234 (46,2)	25 (10,7)	209 (89,3)	2,09 (1)	0,148^a^
Sim	273 (53,8)	41 (15,0)	232 (85,0)		
Renda (reais), n (%)					
Menor que 1.245	169 (31,4)	23 (13,6)	146 (86,4)		
Entre 1.245 e 3.319	244 (45,4)	23 (9,4)	221 (90,6)	9,24 (2)	0,010^a^
Maior que 3.319	125 (23,2)	26 (20,8)	99 (79,2)		
Hipertensão, n (%)					
Não	263 (52,0)	29 (11,0)	234 (89,0)	1,62 (1)	0,203^a^
Sim	243 (48,0)	36 (14,8)	207 (85,2)		
Diabetes, n (%)					
Não	375 (71,6)	43 (11,5)	332 (88,5)	1,53 (1)	0,217^a^
Sim	149 (28,4)	23 (15,4)	126 (84,6)		
Dificuldade para ouvir, n (%)					
Não	344 (63,8)	39 (11,3)	305 (88,7)	3,36 (1)	0,067^a^
Sim	195 (36,2)	33 (16,9)	162 (83,1)		
Zumbido, n (%)					
Não	327 (60,6)	44 (13,5)	283 (86,5)	0,01 (1)	0,917^a^
Sim	213 (39,4)	28 (13,2)	185 (86,8		
Exposição a ruído, n (%)					
Não	326 (60,4)	48 (14,7)	278 (85,3)	1,38 (1)	0,241^a^
Sim	214 (39,6)	24 (11,2)	190 (88,8)		
Uso de prótese auditiva, n (%)					
Não	520 (96,7)	65 (12,5)	455 (87,5)	10,45 (1)	0,001^a^
Sim	18 (3,3)	7 (38,9)	11 (61,1)		
Idade (anos) - média (DP)	63,1 (8,7)	71,3 (8,7)	61,9 (8,0)	60,91 (1)	< 0,001^b^
IMC (kg/m^2^) - média (DP)	28,4 (5,2)	27,0 (4,4)	28,5 (5,2)	5,73 (1)	0,017^b^

DP: desvio padrão; g.l.: graus de liberdade.

Nota: dados faltantes: uso de álcool (75 observações), tabagismo (12 observações), raça (3 observações), dislipidemia (33 observações), renda (2 observações), hipertensão (34 observações), diabetes (16 observações), IMC (19 observações), dificuldade para ouvir (1 observação) e uso de prótese auditiva (2 observações).

Significância estatística. Foi adotado 5% como nível de significância.

^a^ Teste qui-quadrado de Pearson.

^b^ Teste de Mann-Whitney.

**Tabela 3:** Distribuição de frequência conjunta da perda auditiva na melhor/pior orelhaa.

Orelha - critério	Orelha - critério							n (%) de indivíduos com perda auditiva de acordo com cada critério
		Não		Sim		valor-p	Valor da estatística (g.l.)	
		n	%	n	%	(c^2^)		
	Melhor orelha - PTA							Melhor orelha – PTA
Melhor orelha -*speech-frequency* PTA	Não	432	92,7	2	2,7		327,88 (1)	74 (13,7)
	Sim	34	7,3	72	97,3	< 0,001		
	Melhor orelha - PTA							Melhor orelha – *high-frequency* PTA
Melhor orelha - *high-frequency* PTA	Não	290	62,2	2	2,7		91,12 (1)	248 (45,9)
	Sim	176	37,8	72	97,3	< 0,001		
	Pior orelha - PTA							Pior orelha - PTA
Pior orelha - *speech-frequency* PTA	Não	375	89,3	4	3,3		329,52 (1)	120 (22,2)
	Sim	45	10,7	116	96,7	< 0,001		
	Pior orelha - PTA							Pior orelha - *high-frequency* PTA
Pior orelha - *high-frequency* PTA	Não	215	51,2	2	1,7		95,23 (1)	323 (59,8)
	Sim	205	48,8	118	98,3	< 0,001		
	Melhor orelha - *speech-frequency* PTA							Melhor orelha - *speech-frequency* PTA
Melhor orelha - *high-frequency* PTA	Não	292	67,3	0	0,0		155,29 (1)	106 (19,6)
	Sim	142	32,7	106	100,0	< 0,001		
	Pior orelha - *speech-frequency* PTA							Pior orelha - *speech-frequency* PTA
Pior orelha - *high-frequency* PTA	Não	217	57,3	0	0,0		154,11 (1)	161 (29,8)
	Sim	162	42,7	161	100,0	< 0,001		

PTA: médias dos limiares auditivos de tons puros; g.l.: graus de liberdade.

^a^ Distribuição da perda auditiva na melhor/pior orelha: PTA e perda auditiva na melhor/pior orelha – *speech-frequency* - PTA e *high-frequency* – PTA e distribuição de frequência conjunta da perda auditiva na melhor/pior orelha – *speech-frequency* – PTA e perda auditiva na melhor/pior orelha – *high-frequency* – PTA; número (porcentagem) de indivíduos, entre os 540 estudados, que apresentaram perda auditiva na melhor e pior orelha sob PTA, *speech-frequency* PTA e *high-frequency* PTA. Significância estatística. Foi adotado 5% como nível de significância e teste qui-quadrado de Pearson.

### Aspectos Éticos

A presente pesquisa foi aprovada pela Comissão de Ética para Análise de Projetos de Pesquisa do Hospital das Clínicas da Faculdade de Medicina da Universidade de São Paulo (CAAE 15171019.7.0000.0065).

## RESULTADOS

Dentre os 540 indivíduos, houve maior prevalência do sexo feminino (54,4%), idade média de 63,1 anos (8,7) e IMC médio de 28,4 kg/m^2^ (5,2). Em relação à escolaridade, 48,6% possuíam nível superior. Quanto às condições clínicas e audiológicas, 53,8% apresentavam DLP, 48% HAS e 28,4% DM, 70,3% eram usuários de álcool, 36,2% relataram dificuldade para ouvir, 39,4% apresentavam zumbido, 39,6% referiram exposição a ruído e apenas 3,3% utilizavam prótese auditiva. A taxa de falha no teste de equilíbrio foi de 13,3% (n = 72). Na análise bivariada, observou-se associação estatisticamente significativa entre a conclusão no teste de equilíbrio e as variáveis idade (p < 0,001), IMC (p = 0,017), renda (p = 0,010) e uso de prótese auditiva (p = 0,001) (Tabela 1).

A Tabela 2 apresenta a associação entre perda auditiva e a conclusão final do teste de equilíbrio. Houve associação significante entre presença de perda auditiva, tanto na melhor quanto na pior orelha, para os critérios PTA, *speech-frequency* PTA e *high-frequency* PTA e o teste de equilíbrio (p < 0,001 em todos os casos), com maior proporção de falhas no teste de equilíbrio para os indivíduos com perda auditiva.

**Tabela 2:** Distribuição de frequência conjunta da perda auditiva e conclusão final do teste de equilíbrio.

Perda auditiva	Teste de equilíbrio				Valor da estatística (g.l.)	valor-p
	Falhou		Passou			
	n	%	n	%		(c2)
Melhor orelha – PTA						
Não	52	11,2	414	88,8	13,92 (1)	< 0,001^a^
Sim	20	27,0	54	73,0		
Pior orelha – PTA						
Não	43	10,2	377	89,8	15,67 (1)	< 0,001^a^
Sim	29	24,2	91	75,8		
Melhor orelha – *speech-frequency* PTA						
Não	42	9,7	392	90,3	25,57 (1)	< 0,001^a^
Sim	30	28,3	76	71,7		
Pior orelha – *speech-frequency* PTA						
Não	36	9,5	343	90,5	16,18 (1)	< 0,001^a^
Sim	36	22,4	125	77,6		
Melhor orelha – *high-frequency* PTA						
Não	22	7,5	270	92,5	18,50 (1)	< 0,001^a^
Sim	50	20,2	198	79,8		
Pior orelha – *high-frequency* PTA						
Não	15	6,9	202	93,1	12,94 (1)	< 0,001^a^
Sim	57	17,7	266	82,3		

PTA: médias dos limiares auditivos de tons puros; g.l.: graus de liberdade.

^a^ Significância estatística. Foi adotado 5% como nível de significância e teste qui-quadrado de Pearson.

Com relação aos critérios de perda auditiva, verificou-se que, considerando a melhor orelha, a perda auditiva foi identificada em 13,7% dos indivíduos pelo PTA, 19,6% pelo *speech-frequency* PTA e 45,9% pelo *high-frequency* PTA. Na pior orelha, a prevalência de perda auditiva foi de 22,2% pelo PTA, 29,8% pelo *speech-frequency* PTA e 59,8% pelo *high-frequency* PTA (Tabela 3).

Após a aplicação do método *backward* de seleção de variáveis, os seis modelos finais de regressão logística ajustados contaram apenas com a variável idade. Verificou-se que a chance de um indivíduo passar no teste de equilíbrio diminui 11,1% ([1–0,889] x 100%) a cada aumento de um ano na idade (Tabela 5). Vale ressaltar que apesar da idade estar associada às perdas auditivas (Tabela 4), nenhum modelo de regressão logística ajustado acusou multicolinearidade entre idade e perda auditiva.

**Tabela 5:** Resultados do modelo final de regressão logística ajustado.

Termo	Estimativa	Erro padrão	Valor-p	Razão de chances	IC95%
Constante	9,720	1,070	< 0,001^a^		
Idade	-0,118	0,015	< 0,001^a^	0,889	(0,862–0,916)

IC95%: intervalo de confiança de 95%.

^a^ Significância estatística. Foi adotado 5% como nível de significância e modelo de regressão logística múltipla ajustado pelo método *backward*.

**Tabela 4:** Média (desvio padrão) da idade para a perda auditiva na melhor/pior orelha sob o PTA, *speech-frequency* PTA e *high-frequency* PTA.

Perda auditiva	Não	Sim	Valor da estatística (g.l.)	Valor-p
Melhor orelha - PTA	61,7 (7,8)	72,1 (9,2)	68,33 (1)	< 0,001^a^
Pior orelha - PTA	61,3 (7,5)	69,5 (9,6)	65,85 (1)	< 0,001^a^
Melhor orelha - *speech-frequency* PTA	61,1 (7,3)	71,6 (8,9)	97,99 (1)	< 0,001^a^
Pior orelha *- speech-frequency* PTA	60,6 (7,1)	69,2 (9,1)	98,52 (1)	< 0,001^a^
Melhor orelha - *high-frequency* PTA	59,3 (6,5)	67,7 (8,8)	119,92 (1)	< 0,001^a^
Pior orelha - *high-frequency* PTA	58,5 (6,2)	66,2 (8,8)	101,65 (1)	< 0,001^a^

PTA: médias dos limiares auditivos de tons puros; g.l.: graus de liberdade.

^a^ Significância estatística. Foi adotado 5% como nível de significância e teste de Mann-Whitney.

A fim de aprofundar a análise referente a associação entre o desfecho e a presença de perda auditiva para os seis critérios, a idade foi categorizada em quatro faixas etárias (Até 59 anos, de 60 a 69 anos, de 70 a 79 anos e de 80 a 89 anos). Em seguida, para cada faixa etária, foram ajustados seis modelos de regressão logística, um para cada critério de perda auditiva, incluindo as demais variáveis explicativas. Para todos os modelos, após a aplicação do método *backward* de seleção de variáveis, nenhuma variável explicativa mostrou significância.

## DISCUSSÃO

Nossos achados mostraram associação entre perda auditiva e pior desempenho no teste de equilíbrio postural semiestático em adultos e idosos do ELSA-Brasil. Além disso, verificamos que a proporção de falhas no teste de equilíbrio foi significativamente maior para o critério de perda auditiva que incluía as frequências altas no cálculo da média dos limiares auditivos. Além disso, as variáveis explicativas idade, renda e IMC mostraram associação ao pior desempenho no teste de equilíbrio nas análises bivariadas. No entanto, após ajustes, a única variável que manteve associação com o desfecho foi a idade.

Na análise considerando os seis critérios de perda auditiva e o desempenho no teste de equilíbrio, observou-se, para todos eles, maior prevalência de falhas para os indivíduos com perda auditiva. Esses achados estão de acordo com evidências de que perdas auditivas podem impactar a orientação espacial, a detecção de pistas sonoras ambientais e, consequentemente, aumentar o risco de quedas^18^. Isto porque o comprometimento auditivo pode reduzir a capacidade de integração sensorial entre sistemas auditivo e vestibular, prejudicando a estabilidade postural^6^.

No estudo de Lin et al.^18^, utilizando dados do NHANES^15^, foi analisada a relação entre perda auditiva, definida pela média dos limiares audiométricos nas frequências de 0,5; 1; 2 e 4 kHz, e quedas autorreferidas em adultos de 40 a 69 anos. Os resultados mostraram que, para cada aumento de 10 dB na perda auditiva, houve incremento de 1,4 vez na probabilidade de relatar uma queda nos 12 meses anteriores, mesmo após ajuste para múltiplos fatores de confusão.

Em um acompanhamento longitudinal de 10 anos, Jansen et al.^19^ investigaram a associação entre a capacidade de reconhecer a fala em ambientes com ruído (*speech-in-noise*) e quedas recorrentes. A pesquisa incluiu adultos com 40 anos ou mais e demonstrou que o pior desempenho no teste de compreensão de fala em ruído esteve associado a um risco aproximadamente duas vezes maior de quedas recorrentes, especialmente em participantes com obesidade e limiares auditivos mais elevados. Os autores sugeriram que a dificuldade de processar sinais de fala em contextos acusticamente complexos pode comprometer a orientação espacial e aumentar a vulnerabilidade a quedas, reforçando a importância de considerar a função auditiva em frequências relevantes para a percepção de fala em ambientes ruidosos. Além disso, perdas auditivas frequentemente coexistem com disfunções vestibulares e comorbidades crônicas, como DM e doenças cardiovasculares, que podem potencializar a instabilidade postural^20^.

A análise comparativa entre os critérios de perda auditiva mostrou diferentes prevalências de perda auditiva (variando de 13,7% a 59,8%). Diferentes critérios de perda auditiva são utilizados na literatura, com combinações distintas de frequências e/ou orelhas, assim como critérios de corte em diferentes limiares auditivos, o que pode gerar diferentes classificações de perda auditiva, prevalências e magnitudes das associações encontradas^16,21^.

A escolha do critério de perda auditiva deve estar diretamente relacionada aos objetivos e desfechos investigados. Por exemplo, os estudos de Yévenes-Briones et al.^21^e Lin et al.^16^ utilizaram três critérios de perda auditiva, de forma semelhante ao presente estudo, permitindo avaliar diferentes dimensões da audição. O PTA é utilizado para representar a sensibilidade auditiva nas frequências médias, enquanto o *speech-frequency* PTA acrescenta a frequência de 4.000 Hz, que é importante para a inteligibilidade da fala. Já o *high-frequency* PTA concentra-se em frequências mais altas, frequentemente as primeiras a serem afetadas em perdas auditivas relacionadas ao envelhecimento ou induzidas por ruído^22^.

Putter-Katz et al.^23^ e Schubert et al.^24^ identificaram associação entre perda auditiva e instabilidade postural. Putter-Katz et al.^23^ demonstraram que perdas auditivas em altas frequências contribuem para maior instabilidade e risco de quedas, sugerindo que a degradação da via auditiva afeta os mecanismos compensatórios do equilíbrio. Já Schubert et al.^24^ apontaram que a perda auditiva, independentemente da faixa de frequência, está relacionada a piores escores em testes vestibulares e de marcha, reforçando a hipótese de que a audição atua como componente sensorial adicional no controle postural. Esses achados reforçam a importância da avaliação auditiva, incluindo as frequências altas nos critérios de classificação de perda auditiva, quando o objetivo é avaliar o equilíbrio e o risco de quedas em idosos.

Por isso, no caso do presente estudo, que avaliou adultos mais velhos e idosos, utilizamos critérios que refletissem a perda auditiva relacionada à idade, que se inicia pelas frequências mais altas^25,26^ e, de fato, verificamos prevalências mais altas de perda auditiva para o critério *high-frequency* PTA, assim como, também para este critério, houve maior proporção de falhas no teste de equilíbrio.

Quanto à associação entre o desempenho no teste de equilíbrio e as variáveis explicativas, incluindo a perda auditiva, bem como as variáveis sociodemográficas e clínicas, após a aplicação do método *backward* de seleção de variáveis, os modelos finais de regressão logística ajustados contaram apenas com a variável idade, indicando que a idade foi a única que mostrou significância, assim como a chance de um indivíduo passar no teste de equilíbrio diminui 11,1% a cada aumento de um ano na idade. Sabe-se que o envelhecimento acarreta num declínio progressivo das capacidades físicas e funcionais, repercutindo diretamente no equilíbrio e na mobilidade, o que pode diminuir o desempenho funcional no equilíbrio e elevar o risco de quedas nessa população. Vermisso et al.^27^ avaliaram o estado funcional e a capacidade de equilíbrio em idosos saudáveis e encontraram associação entre equilíbrio e estado funcional em idosos, de modo que déficits em um refletem no outro. Outros autores^28,29^enfatizaram que o declínio no estado funcional devido ao envelhecimento pode ser atribuído a alterações no equilíbrio.

Deve-se mencionar que, inicialmente, na análise bivariada, além da idade e da perda auditiva, as variáveis renda e IMC mostraram associação com o desfecho. No entanto, nas análises ajustadas, a única que manteve a associação com o equilíbrio foi a idade.

Estudos prévios^30,31^ verificaram que a baixa renda e a menor escolaridade estiveram associadas às quedas em idosos, sugerindo influência de condições sociodemográficas na vulnerabilidade dessa população. Esses resultados indicam que grupos diferentes podem ser afetados de maneira desproporcional quando se trata de quedas/ estabilidade postural; sendo assim, tais variáveis devem ser levadas em consideração neste tipo de estudo, assim como na comparação entre diferentes estudos. Importante enfatizar que a amostra ora avaliada é diferenciada, já que todos são servidores públicos ativos ou aposentados e, portanto, com melhores condições financeiras e mais acesso a serviços de saúde do que a população brasileira em geral^13^. Além disso, quase metade dos participantes possuía ensino superior, proporção que está muito acima da média nacional^32^. Conforme sugerido em estudo prévio^33^, a maior escolaridade pode trazer maior conhecimento sobre saúde, hábitos de vida mais saudáveis e acesso mais oportuno a serviços de saúde.

Em relação ao IMC, a associação verificada inicialmente na análise bivariada foi entre menor IMC e falha no teste de equilíbrio. Estudo prévio^34^ verificou um IMC mais elevado associado a menor chance de quedas em um grupo de idosas e os autores sugeriram que uma maior quantidade de tecido corporal poderia exercer um efeito protetor, seja pelo maior amortecimento em casos de impacto ou pela relação positiva com a massa magra, que é importante para a preservação da capacidade funcional. Contrariamente, outro estudo^35^ observou que indivíduos obesos apresentaram um risco maior de quedas em comparação àqueles com peso saudável, destacando que os primeiros apresentaram maior prevalência de doenças crônicas, uso de múltiplos medicamentos e pior qualidade de vida. Um terceiro estudo^36^ressaltou que o baixo IMC, que é marcador de desnutrição em idosos, esteve fortemente associado a maior risco de quedas e fraturas, sobretudo em razão da perda de massa magra e da sarcopenia. Assim, os autores enfatizaram que, em idosos, pontos de corte mais elevados de IMC (24 kg/m^2^–27 kg/m^2^), quando comparados a adultos mais jovens, são considerados adequados e podem exercer um papel protetor^36^.

Visando aprofundar as análises entre desempenho no teste de equilíbrio e perda auditiva, considerando que a única variável que manteve associação com o desfecho foi a idade, categorizamos tal variável em quatro faixas etárias e, para cada uma delas, foram ajustados os seis modelos de regressão logística, um para cada critério de perda auditiva, que incluíram as demais variáveis explicativas pelo método *backward*. Contudo, não houve significância estatística para nenhuma das variáveis explicativas, inclusive para perda auditiva, em nenhum dos modelos, confirmando que o desfecho é explicado apenas pela variável idade. Este achado enfatiza o peso da variável idade, também no desempenho do equilíbrio, se sobrepondo às demais variáveis, semelhante ao verificado para perda auditiva^16^. Uma possível explicação para estes resultados é que algumas destas variáveis (sociodemográficas e clínicas) podem apresentar uma associação mais fraca (como verificado nas primeiras análises do presente estudo), mas seus efeitos são mascarados por fatores de risco mais fortes, como a idade^16^.

Numa revisão sistemática e metanálise sobre o tema, os autores enfatizam que não fica claro se existe associação causal real entre perda auditiva e alterações no equilíbrio ou apenas a coexistência de alterações relacionadas à idade, que leva ao comprometimento de ambos os sistemas. O estudo verificou que a perda auditiva relacionada à idade esteve associada a comprometimento do equilíbrio e da mobilidade; contudo, os autores destacaram que parte do déficit observado refletiu fatores de confusão residuais relacionados à idade. Assim, sugeriram que, embora a idade cronológica continue sendo um fator determinante nos resultados relacionados ao equilíbrio, a perda auditiva relacionada à idade contribui com um risco adicional e modificável por meio de vias sensoriais e cognitivo-motoras, e que a reabilitação pode oferecer ganhos clinicamente significativos no controle postural dentro de estratégias multimodais de prevenção de quedas^37^.

Como limitações do estudo, devem ser levados em conta alguns aspectos. Primeiramente, o fato de a amostra estudada estar inserida no ELSA-Brasil deve ser considerado, visto que se trata de um grupo com maior acesso aos serviços de saúde, maior conscientização quanto ao cuidado com a própria saúde e, possivelmente, melhor adesão a práticas preventivas, o que pode ter influenciado nos desfechos observados. Ademais, devido ao delineamento transversal, não é possível estabelecer relações de causalidade ou temporalidade entre perda auditiva e desempenho no equilíbrio. Deve-se mencionar também que o uso exclusivo do teste de Romberg modificado, para avaliar o equilíbrio, não substitui avaliações instrumentais mais detalhadas e, portanto, limita a avaliação de componentes mais finos do controle postural, o que pode atenuar a identificação de associações mais sutis.

Dentre as potencialidades do presente estudo, destaca-se o uso de uma amostra numerosa, proveniente do ELSA-Brasil/ELSA-A, que faz uso de protocolos padronizados e de exames clínicos e laboratoriais objetivos (incluindo a avaliação audiológica e a triagem do equilíbrio), o que fortalece a validade dos resultados obtidos. Além disso, o presente estudo apresenta caráter inovador no contexto brasileiro.

Em síntese, a idade mostrou-se como principal variável associada ao desempenho no equilíbrio postural em adultos e idosos do ELSA-A. A perda auditiva apresentou associação com o equilíbrio, particularmente quando consideradas as frequências altas. Variáveis sociodemográficas e clínicas, como renda e IMC, parecem influenciar o desempenho no equilíbrio de forma mais complexa e indireta. Esses achados reforçam a necessidade de abordagens integradas e multifatoriais na prevenção de quedas, incorporando a avaliação auditiva e a triagem do equilíbrio ao cuidado em saúde de adultos e idosos.

## Data Availability

Os dados estão disponíveis mediante solicitação ao autor correspondente.
